# Report of a Rare Case of Computed Tomography Diagnosis of Hydrocarbon Pneumonitis

**DOI:** 10.7759/cureus.19914

**Published:** 2021-11-26

**Authors:** Parag S Mahajan, Jouhar J Kolleri, Hanan Farghaly

**Affiliations:** 1 Clinical Imaging, Hamad Medical Corporation, Doha, QAT; 2 Laboratory Medicine and Pathology, Hamad Medical Corporation, Doha, QAT

**Keywords:** diesel siphonage, chest x-ray, hydrocarbon pneumonitis, diagnosis, ct (computed tomography) imaging

## Abstract

The practice of manually siphoning diesel from fuel tanks is widespread among car mechanics in Asian countries. To date, there are just a few reports in the literature about hydrocarbon pneumonitis caused by diesel fuel aspiration. An early diagnosis based on clinical suspicion, imaging results, and histopathology can help prevent permanent damage to the lungs.

## Introduction

Manual fuel siphonage is the process of moving fluid from one container to another to fill fuel tanks, which is highly hazardous and could result in serious repercussions. Hydrocarbon pneumonitis is an acute chemical pneumonitis caused by the aspiration of diesel fuel. It is imperative to be aware of symptoms and radiological findings of hydrocarbon pneumonitis since these can be diagnostic in the correct clinical scenario. We present a case of a patient with severe hydrocarbon pneumonitis after fuel siphonage.

## Case presentation

A 49-year-old gentleman came to the emergency department with a four-day history of fever and cough, which started after accidental aspiration of diesel during siphonage of a motor vehicle. He also had a one-day history of shortness of breath and pleuritic-type chest pain. There was no history of wheeze or altered sensorium. No history of allergy, recent travel, and contact with sick patients or animals was noted. On examination, he was conscious, oriented, and not in distress. His oral temperature was 38 degrees Celsius, heart rate was 95 bpm, respiratory rate was 20/minute, blood pressure was 121/71 mm Hg, and oxygen saturation was 99%. There was no cyanosis. On respiratory examination, normal breathing sounds, percussion notes, and vocal and tactile resonance were noted. No added sounds were heard on auscultation. All other systemic examinations were within normal limits. Laboratory investigations showed elevated inflammatory markers. Baseline hemogram and renal and liver function tests were normal. Arterial blood gas analysis and electrocardiogram were normal.

**Figure 1:**
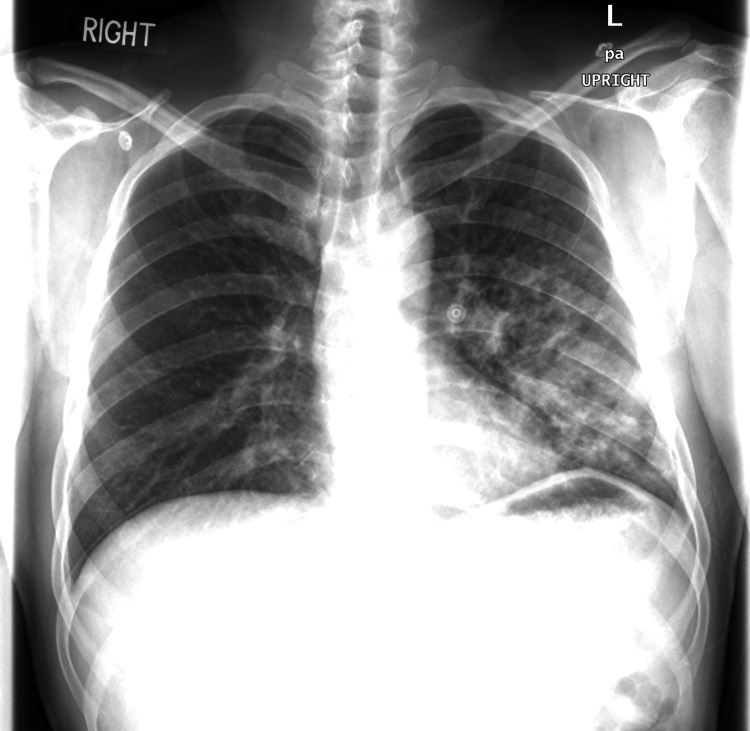
Chest X-ray showing parenchymal lung opacities in left mid and lower zones.

Chest X-ray showed patchy parenchymal haziness/ground glass lung opacities in the left mid and lower zones, keeping with infection-related pneumonitis (Figure 1). In addition, repeated chest X-rays showed progression of the lung consolidation in the left middle and lower zones.

COVID-19 reverse transcription polymerase chain reaction (RT-PCR) test was performed on presentation to the emergency department, and came as negative. Diagnosis of infective pneumonia was made on the day of admission, and antibiotic treatment started. When his fever did not subside, and repeated chest X-rays showed no resolution of lung opacities, a diagnosis of non-resolving pneumonia was made. He was investigated further to rule out pulmonary tuberculosis. Quantiferon test was indeterminate. Two sets of acid-fast bacilli sputum smear and tuberculosis polymerase chain reaction came negative. Likewise, two sets of blood culture were also negative.

**Figure 2:**
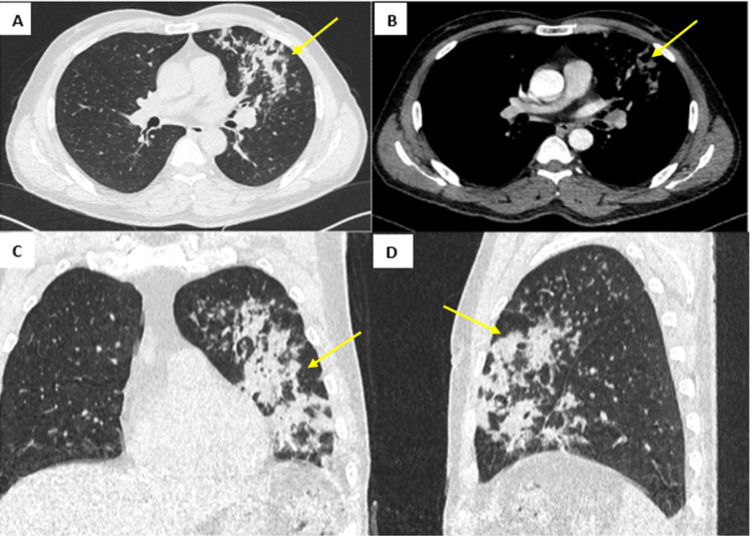
Computed tomography scan of chest with intravenous contrast, lung window A) axial, C) coronal and D) sagittal cuts, showing patchy areas of consolidation in the left lung. B) Axial soft tissue window showing focal lesions with central non-enhancing hypodensity, suggestive of low-density consolidation in the left hilum.

For further investigation, a CT (computed tomography) scan of the chest with contrast was requested. CT scan showed patchy areas of consolidation in the left lung, predominantly in the lingula and right middle lobe, with centrilobular opacities in the left lung (predominantly in the left upper lobe) and the right upper lung lobe. In addition, focal lesions with central non-enhancing hypodensity in the left hilum were suggestive of low-density consolidation rather than lymphadenopathy (Figure 2). In view of the history of diesel aspiration, these features suggested a possibility of hydrocarbon/chemical pneumonitis secondary to aspiration of diesel.

**Figure 3:**
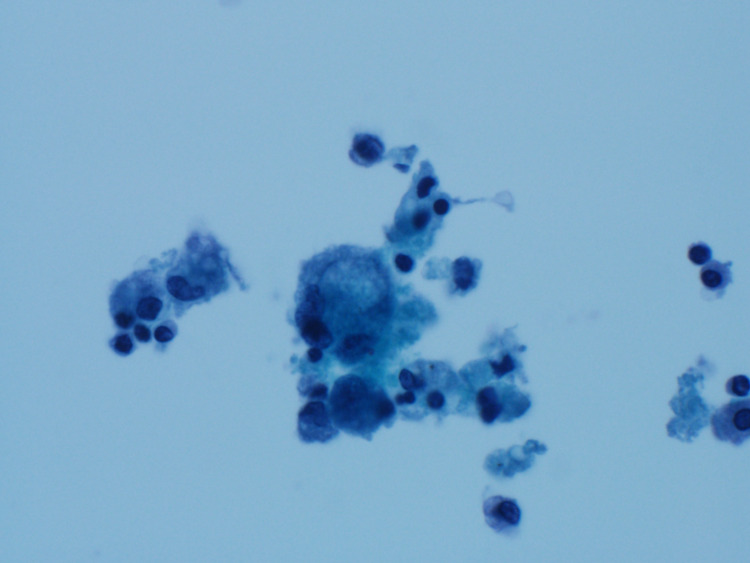
Bronchoalveolar lavage fluid cytology showing lipid-laden macrophages with lipid droplets/vacuoles within.

Bronchoscopy done to exclude endobronchial tuberculosis and fungal infection showed no evidence of endobronchial lesions or endobronchial tuberculosis. Bronchial biopsy was negative for malignancy and granulomas. Bronchoalveolar lavage (BAL) demonstrated lipid-laden macrophages (Figure 3), which further confirmed the radiological diagnosis of hydrocarbon pneumonitis.

He was given intravenous Augmentin 1200 mg infusion, three times daily for three days and oral naproxen 500 mg, twice daily for five days, got symptomatically better (although chest X-ray showed non-resolving lung opacities), hence discharged. The patient was instructed to follow up regularly in the outpatient clinic and is currently doing well.

## Discussion

Laughlin defined lipoid pneumonia in 1925 as an uncommon kind of lung disease caused by fat-containing products accumulating in the distal airways and alveoli, causing an inflammatory reaction that prevents gas exchange. It is classified as exogenous and endogenous lipoid pneumonia depending on the source of lipids [1,2]. Hydrocarbon pneumonitis following diesel aspiration is a kind of exogenous lipoid pneumonia. Aspirated diesel quickly enters the alveoli without causing a substantial cough yet triggers a strong inflammatory reaction in the lungs. This scenario is rarely reported, despite diesel siphoning from vehicles and fuel tanks being frequent in Asian countries [3].

Hydrocarbons are not absorbed in the airways once aspirated and reach alveoli quickly. They cause alveolar edema, tissue injury, and surfactant degradation [4]. These pathologic alterations come from macrophage activation and inflammatory cytokine release [5]. Electron microscopy shows all characteristics of macrophage activation [6]. The host reaction to inhaled lipid compounds varies in severity.

The most common presentation is fever and breathlessness. Gondouin et al. found that 39% of patients had a fever, and 34% lost weight [7]. In most patients, auscultation of the lungs is normal, but may show crepitations or wheezes. Acute hydrocarbon poisoning has been observed after motor fuel aspiration following siphonage. It can also affect the central nervous system, digestive system, and lungs [8].

Investigation of choice is high-resolution CT characterized by exogenous lipoid pneumonia-like consolidation with crazy-paving pattern. Bronchoscopy can confirm the diagnosis of lipoid pneumonia caused by hydrocarbons, which is identified by foamy macrophages in the alveoli and interstitial spaces [9]. This was well demonstrated in our patient.

As hydrocarbons elicit little cough and can be aspirated directly into the lower lobes, bilateral basal involvement is the most common radiological picture following aspiration [10]. In most cases, radiological findings are out of proportion to the clinical findings [11]. According to Bentacourt et al., the middle lobe is commonly involved in hydrocarbon pneumonitis. In our case, lung opacities were present in the lingula, right middle lobe, and upper lobes [2].

Antibiotic treatment should be started when a patient has a fever, increased inflammatory markers, and tachypnea, even though prophylactic antibiotic treatment is not recommended [12]. Literature shows different treatments, including supportive care (92%), intravenous antibiotics (57.5%), steroids (57.5%), and BAL (32.5%). The use of antibiotics to treat hydrocarbon pneumonitis is inefficient. Since it is impossible to tell the difference between hydrocarbon pneumonitis and a superimposed lung infection using radiological findings, most patients with hydrocarbon pneumonitis are treated with antibiotics [13]. Patients with hydrocarbon pneumonitis had a good response to steroid therapy, according to Sen et al. [14]. The use of corticosteroids and antibiotics to treat hydrocarbon aspiration was investigated in an animal trial, but the results showed little benefit [15]. In a few cases, patients with hydrocarbon pneumonitis saw substantial improvements after BAL [16,17]. Hydrocarbon pneumonitis rarely leads to serious morbidity or mortality in patients with good supportive care. However, it would take two weeks to eight months for the radiological findings to get resolved after clinical improvement [18].

Our patient was treated with intravenous Augmentin infusion and oral naproxen and started showing improvement after one week of starting treatment. After the CT diagnosis of hydrocarbon pneumonitis, since the cytological confirmation was not available and he was diabetic, the treating physician considered a higher possibility of it being a fungal infection than chemical pneumonitis. This was the reason for not starting steroid treatment. This also shows the need to educate the physicians including the radiologists about this rare condition.

## Conclusions

When a mechanic or garage worker has a history of diesel siphonage and develops corresponding respiratory symptoms, a diagnosis of diesel siphoner's lung or hydrocarbon pneumonitis should be contemplated. Early management can benefit from a high index of clinical suspicion backed up by radiological evidence and histopathology, which can further prevent permanent lung damage. The mainstay of treatment is supportive care, with antibiotics, steroids, and BAL as appropriate therapeutic options.
